# The Brain Vulnerability Index: Development and Validation of a Machine Learning–Derived, Community-Informed Geospatial Risk Score for Cognitive Impairment

**DOI:** 10.21203/rs.3.rs-9381770/v1

**Published:** 2026-04-27

**Authors:** Yusuf Tamer, Autumn Noon, Zhiyi Yang, Kexin Yu, Amil Shah, Albert Karam, Ihab Hajjar

**Affiliations:** Parkland Center for Clinical Innovation; Parkland Center for Clinical Innovation; University of Texas Southwestern Medical Center; University of Texas Southwestern Medical Center; University of Texas Southwestern Medical Center; Parkland Center for Clinical Innovation; University of Texas Southwestern Medical Center

## Abstract

**Background:**

Social determinants of health (SDOH) are increasingly recognized as important contributors to cognitive impairment, including Alzheimer’s disease and related dementias. Existing indices are heavily weighted toward financial indicators and are not validated against cognitive outcomes. We aimed to develop and validate a novel brain-specific SDOH index that identifies high-risk communities for cognitive impairment.

**Methods:**

The Brain Vulnerability Index (BVI) integrates patient-level electronic health record data with neighborhood-level SDOH from the Community Vulnerability Compass, a population health analytics framework. In the model development phase, electronic health record data were obtained from patients seen at Parkland Health (Dallas, TX). Cases were defined as individuals with at least one ICD-10 diagnosis suggestive of cognitive impairment from 2015 to 2023 and controls were age-matched with no ICD-10 diagnosis. External validation against serial cognitive performance measured by the Montreal Cognitive Assessment (MoCA) and clinically adjudicated consensus diagnosis of cognitive impairment of any etiology was performed in community-based (Dallas Heart Study) and clinically based (Alzheimer’s Disease Research Center) cohorts.

**Results:**

The model development sample included 39,570 cases and 192,060 controls. Derived BVI at the block group level achieved detection of electronic health documentation suggestive of cognitive impairment at a balanced accuracy of 54.6%. In external validation (n = 1,395), higher BVI values were associated with lower MoCA scores (β = −0.35; p = 0.009) and with faster cognitive decline (− 1.04 vs − 0.42 MoCA points/year in high- vs low-risk groups; p < 0.0001). BVI was also associated with adjudicated cognitive impairment in the community-based cohort only (p = 0.04). Across analyses, BVI correlated with but outperformed existing neighborhood indices.

**Conclusions:**

BVI, a novel multidimensional brain-specific SDOH index, is clinically anchored and is externally validated. It may assist in earlier identification of at-risk communities with accelerated cognitive decline and aid in resource allocation for targeted prevention strategies.

## Background

Alzheimer’s disease (AD) and related dementias (ADRD) are neurodegenerative diseases with AD being the fifth-leading cause of death in the US.^1,2^ AD impacted an estimated 6.9 million Americans 65 and older in 2024 and is expected to affect 13.8 million Americans by 2060.^3^ The percentage of Americans with AD increases with age: 5.0% of people aged 65–74, 13.2% of people aged 75 to 84, and 33.4% of people 85 and older.^1,2^ Despite great advances in our understanding of ADRD, nearly half of cognitive impairment risk remains unexplained.^4^ More recently, risk models suggest a strong contribution of non-medical drivers to cognitive impairment risk.^5^

Identifying elevated risk groups, especially beyond traditional risk factors, improves efficiencies of prevention programs as well as case ascertainments and identifying those that would benefit most from therapeutic programs. Increasingly, social determinants of health (SDOH), and more broadly non-medical drivers of health (NMDOH), have been identified as important, potentially modifiable risk factors for AD and ADRD.^6–8^ Currently available measures of SDOH are not brain-specific, emphasize financial and structural vulnerability, and are rarely operationalized as patient-level tools due to the lack of interpretable thresholds for use in clinical practice.^9,10^ For example, the Area Deprivation Index (ADI) is widely used to quantify neighborhood socioeconomic disadvantage in ADRD research, However, it provides generic, ranking of areas masking within-neighborhood heterogeneity and lacks calibration as an individual-level brain health risk tool.^11,12^ To achieve progress towards clinical implementation, we need new tools encompassing a wide variety of measures that are highly relevant to ADRD and that identify patient-level risk. One emerging approach is to use machine learning (ML) and other artificial intelligence (AI) methods that jointly analyze high-dimensional clinically and non-clinically derived data, including a wide range of SDOH indicators, to identify an individual’s risk of cognitive impairment.^13^ Fragmented diverse social data sources lacking clinical context have hindered the creation of more advanced SDOH indices.

The Parkland Center for Clinical Innovation (PCCI), a North Texas nonprofit specializing in data-driven solutions, developed the Community Vulnerability Compass (CVC) to bridge the gap between socioeconomic data and medical records. Through its unique partnership with Parkland Health, PCCI integrates deep clinical datasets with various public and private sources. This allows for the sophisticated ‘deep phenotyping’ of SDOH within a clinical context, creating a more accurate picture of patient vulnerability. Aligned with the Healthy People 2030 SDOH framework, the CVC integrates expansive state and national datasets to characterize SDOH at the census-block group level.^14^ By bridging this infrastructure with electronic health record (EHR) data from a safety-net hospital, PCCI overcomes previous limitations in SDOH measurement. This linkage creates a multidimensional framework capable of generating actionable insights anchored in disease-specific clinical outcomes to characterize individual- and neighborhood-level SDOH.^15^ This approach has been applied to identify risk indices for other conditions.^16,17^ Here we apply similar methodology to clinically identified diagnoses suggestive of cognitive impairment.

Therefore, we aimed to leverage CVC’s data infrastructure linked with Parkland Health EHR data and advanced ML methods to develop the Brain Vulnerability Index (BVI), a multidimensional measure reflecting individual SDOH-related risk for clinically identifiable cognitive impairment. We then sought to validate BVI’s associations with cognitive performance and impairment across clinical- and population-based cohort studies.

## Methods

This study employed a two-stage hybrid design. In Stage 1 (BVI Derivation), a retrospective case-control study was conducted using Parkland Health EHR data, integrated with census block group–level SDOH data from diverse national datasets, to develop and internally validate the BVI. In Stage 2 (External Validation), BVI performance was evaluated in two independent and geographically overlapping cohort studies in the Dallas–Fort Worth (DFW) metropolitan area. Both cohort studies were approved by the UT Southwestern Institutional Review Board, and written informed consent was obtained from all participants in the validation cohorts.

### Data used for BVI development:

Clinical data were extracted from Parkland Health’s EHR, covering the period November 1, 2015, through November 30, 2023. Parkland Health is a 900-bed safety-net hospital with an integrated network of community-based primary care clinics serving the DFW metropolitan area. Cases were defined as patients with at least one of the following: a documented International Classification of Diseases, Ninth or Tenth Revision (ICD-9/ICD-10) code for mild cognitive impairment, dementia, or a related cognitive disorder; a corresponding Systematized Nomenclature of Medicine – Clinical Terms (SNOMED CT) designation; or a dispensed prescription for ADRD pharmacotherapy (donepezil, rivastigmine, galantamine, memantine, or anti-amyloid monoclonal antibodies), consistent with previously described EHR- approaches.^18,19^ The complete ICD-10 and SNOMED CT code inventory is provided in **Supplementary Table S1**. For each identified case, up to five controls were frequency-matched within the same 10-year age stratum and residing in the same metropolitan statistical area, yielding an approximate 1:5 case-to-control ratio. Extracted EHR variables included: sociodemographic characteristics (age, sex, race/ethnicity, smoking status, and marital status); comorbidity burden, quantified using the Charlson Comorbidity Index (CCI);^20^ and health care utilization in the 12 months preceding the index date (number of primary care, emergency department, and neurology encounter, hospitalizations, and mode of arrival to the healthcare system).

All case and control patients were geo-linked to their corresponding U.S. Census block group using their residential address on record during the study period. For each block group, multidimensional SDOH data were obtained from the CVC.^16,17^ The CVC integrates data from eight authoritative national and local datasets: the American Community Survey;^21^ the Center for Neighborhood Technology Housing and Transportation Index,^22^ Environmental Protection Agency National Walkability Index,^23^ United States Small-Area Life Expectancy Estimates Project,^24^ Program for the International Assessment of Adult Competencies Literacy,^25^ Air pollution from OpenWeather,^26^ Greenspace from ParkServe,^27^ and neighborhood safety data from Applied Geographic Solution’s Crimerisk.^28^ For model development, 26 SDOH indicators derived from these sources were retained and organized into four theoretically informed thematic domains: (1) *Household Essentials* (food security, health insurance coverage, income predictability, and household income); (2) *Good Health* (neighborhoodlevel prevalence of alcohol use disorder, chronic disease burden, cancer burden, life expectancy, and mental health status); (3) *Equitable Communities* (affordable housing availability, air quality, greenspace access, unemployment rates, and neighborhood safety); and (4) *Empowered People* (broadband connectivity, walkability, vehicle access, and adult literacy).^15^

#### Data used for external validation of BVI

Two cohort studies enrolling participants residing within the BVI model development geographic area were selected for external validation. The *Dallas Heart Study and Mind Study* is an extension of the multiethnic Dallas Heart Study (DHS), an ongoing population-based probability sample recruited from 1999–2000 that has been described in detail.^29–43^ DHS serves as the community-based (population-representative) validation cohort. In the 2021–2024 DHS follow-up wave, participants underwent standardized cognitive assessment using the Montreal Cognitive Assessment (MoCA)^44^ as well as a supplementary cognitive battery. Participants were classified as either cognitively unimpaired or cognitively impaired based on aggregate performance across all administered assessments, independent of impairment severity or etiology.

The *Alzheimer’s Disease Center (ADC) at UT Southwestern* served as the clinical validation cohort. ADC participants underwent standardized global cognitive assessment using the MoCA or Mini-Mental State Examination (MMSE), supplemented by a comprehensive neuropsychological battery. Cognitive status was adjudicated using a consensus diagnosis process and classified dichotomously as impaired or unimpaired, independent of the degree or underlying etiology of impairment. For participants assessed with the MMSE, scores were converted to MoCA-equivalent values using the validated Roalf conversion equation (MoCA = 1.13 × MMSE − 6.45), which has demonstrated strong concordance between the two measures across the cognitive spectrum.^45^. A local validation study in this population confirmed a concordance coefficient of 0.85 (p < 0.001).^46^ The ADC sample contributed serial MoCA measurements (median: 3 assessments per participant), enabling longitudinal trajectory analysis.

## Statistical Methods

### Model Development and BVI Derivation.

A supervised binary classification model was employed to generate clinically anchored, outcome-referenced risk scores from CVC-derived SDOH indicators, which were subsequently aggregated into a neighborhood-level brain vulnerability measure. All features were standardized to Z-scores prior to modeling to ensure harmonized scaling across heterogeneous data sources. To account for the potential multicollinearity among SDOH indicators, Least Absolute Shrinkage and Selection Operator (LASSO) regularization was applied for feature selection, with the regularization parameter λ optimized via cross-validation. Class imbalance inherent to the case-control design was addressed using the Synthetic Minority Oversampling Technique (SMOTE).^47^ The final model was specified as regularized logistic regression. Data were partitioned into training (75%) and held-out internal validation (25%) sets using stratified random splitting to preserve class proportions.

Model performance was characterized using macro-averaged F1-score, precision, recall, and balanced accuracy, defined as (sensitivity + specificity) / 2, metrics prioritizing stable contributions suitable for index derivation rather than discrimination performances. The latter were calculated on the held-out validation set including area under the receiver operating characteristic (ROC) curve (AUC),^48^ positive predictive value (PPV),and negative predictive value (NPV). Given that the primary analytical objective was the derivation of a geospatial neighborhood-level index, a moderate individual-level AUC was anticipated and is consistent with established methodology for area-based SDOH indices.^49,50^

Global feature contributions were characterized using log-odds coefficients (b) from the final regularized logistic regression model. The sign and magnitude of each coefficient (b) reflect the relative contribution of each feature to the model-derived risk score underlying BVI construction, rather than indicating absolute or independent risk of cognitive impairment. Feature-level contributions to individual predictions were further quantified using Shapley Additive Explanations (SHAP) analysis applied to a randomly selected subsample of 10,000 training observations.^51^ To derive our geospatial index BVI, individual patient-level risk scores were aggregated to the census block group level by computing the median risk score across all patients attributed to each block group. This median value constitutes the BVI score, which was subsequently advanced to external validation.

### External Validation Analyses.

In the index-building context, validation approaches included construct validity, external criterion validity (associations with cognitive outcomes), and incremental validity (superior performance vs. comparator indices). BVI scores were assigned to validation cohort participants by linking each participant’s residential address at the time of cognitive assessment to the corresponding census block group using the U.S. Census Bureau’s Geocoder (https://geocoding.geo.census.gov/geocoder). Each participant received the model-derived BVI score for their matched block group. For construct validity assessment, two established geospatial vulnerability indices were calculated for each participant: the ADI, a composite measure of neighborhood socioeconomic disadvantage derived from census-based variables including income, educational attainment, employment, and housing quality, with higher scores reflecting greater deprivation;^52^ and the Social Vulnerability Index (SVI), a CDC-developed composite indicator characterizing a community’s susceptibility to adverse health outcomes from external stressors, constructed from census tract–level data on socioeconomic status, household composition, minority status, language barriers, housing type, and transportation access.^53^ Construct validity was assessed by examining the Pearson and Spearman correlations between BVI and each comparator index. To assess whether BVI explained variance in MoCA scores beyond established ADI and SVI, a hierarchical general linear model fit within the combined cohort.

Descriptive statistics are reported as mean (± standard deviation) for continuous variables and as frequency (percentage) for categorical variables. Betweengroup comparisons were performed using independent samples t-tests and chi-squared tests, as appropriate. Model development was implemented in R version 3.5.0 within Azure Databricks Runtime 15.4 (glmnet^54,55^, randomForest^56^ e1071^57^, and pROC^48^), and Python 3.11 in Azure Databricks Runtime 16.4 (scikit-learn^23^, imbalanced-learn^24^ and pandas packages^25^). External validation was conducted in the combined sample and stratified by cohort (ADC and DHS). Cross-sectional associations between BVI and cognitive function were examined using generalized linear models (GLM) for continuous MoCA scores and binary logistic regression for dichotomous cognitive impairment status. Models were specified as unadjusted (BVI only, or BVI + cohort indicator in combined analyses) and multivariable adjusted (additionally controlling for age, sex, and, in combined analyses, cohort), given that these variables were not incorporated into the BVI model development. Effect sizes were quantified using Cohen’s d. Longitudinal cognitive trajectories were examined using linear mixed-effects models with random intercepts for participants to account for within-subject correlation across repeated assessments.

An optimal BVI risk stratification threshold for predicting accelerated cognitive decline was identified using Classification and Regression Tree (CART) analysis.^58^ The CART algorithm recursively partitioned the sample based on BVI values to identify the split point that minimized within-group variance in longitudinal MoCA scores. This data-driven approach identified a threshold of BVI ≥ 0.563. Linear mixed-effects models with random intercepts were subsequently fit to test the hypothesis that participants residing in high-risk neighborhoods (BVI ≥ 0.563) experienced significantly greater rates of cognitive decline compared to those in lower-risk neighborhoods, with the Index × Visit interaction term as the primary parameter of interest.

Construct validity of the BVI was assessed by computing Pearson product-moment correlations between block group–level BVI scores and corresponding SVI and ADI values. Comparative predictive performance across indices was evaluated by contrasting standardized regression coefficients (β), model coefficients of determination (R^2^), and AUC values in parallel cross-sectional and longitudinal models. All statistical tests were two-tailed, with a significance threshold of α = 0.05.

## Results

### Model Development Study Sample:

The BVI derivation cohort comprised 231,630 patients with at least one encounter at Parkland Health during the study period, of whom 39,570 (17.1%) met criteria for EHR-documented cognitive impairment (cases) and 192,060 (82.9%) served as matched controls. Those with EHR based cognitive impairment diagnoses differed significantly from controls in all demographics, social habits, and across all sociodemographic and SDOH domains (all p < 0.001; Table 1). They had less primary care visits, emergency encounters but more mental health and neurology encounters (all p<0.001). **Table S2** in supplementary materials summarizes the differences between impaired and unimpaired groups across all 4 thematic domains and 26 features included in this analysis.

### Model performance and BVI scores:

**Figure 1:**
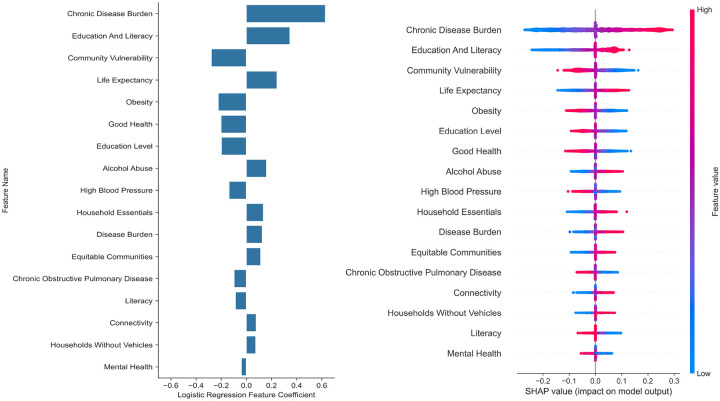
Feature Contributions to Brain Vulnerability Index Predictions. Source: Authors’ own analyses. SHAP (SHapley Additive exPlanations) values for individual features from LASSO-regularized logistic regression model predicting EHR-documented cognitive impairment (n=10,000 subsample). Horizontal position indicates direction and magnitude of feature impact on model output—right (positive SHAP) increases predicted risk, left (negative SHAP) decreases risk. Bar length shows mean absolute SHAP value (feature importance ranking). Color intensity reflects feature value (blue=low, red=high). Chronic disease burden exhibits highest impact (widest SHAP distribution), followed by education/literacy. Negative contributions (community vulnerability, obesity) reflect model-derived protective effects within CVC framework.

Of the 26 CVC-derived SDOH indicators entered into LASSO-regularized logistic regression, 17 features were retained in the final model (**Table S3**; Figure 1). Neighborhood-level chronic disease burden emerged as the dominant feature in the derived index (logistic regression coefficient b = 0.627) and the highest SHAP value, indicating that residence in areas with elevated chronic disease prevalence most strongly increased the predicted probability of cognitive impairment. Education and literacy was the second-ranked feature (β = 0.346), followed by life expectancy risk (β = 0.244) and alcohol abuse burden (β = 0.160); all with positive coefficients, reflecting their impact of an increased risk for cognitive impairment. Conversely, community vulnerability (β = −0.278), obesity prevalence (β = −0.224), overall good health index (β = −0.201), education level (β = −0.199), and high blood pressure burden (β = −0.138) carried negative coefficients, suggesting that these features, as operationalized within the CVC framework, exerted inverse or compensatory contributions to the model output. The directionality of individual features is further illustrated in the SHAP summary plot (Figure 1), which depicts both the magnitude and direction of each feature’s contribution to individual-level predictions, with chronic disease burden demonstrating the widest distribution of SHAP values and the clearest separation by feature value.

On the 25% held-out validation set, the model achieved macro-averaged F1-score = 0.50, precision = 0.50, recall = 0.49, balanced accuracy = 54.6%, NPV = 86.1%, and AUC = 0.57. Balanced accuracy above chance and high NPV confirm stable, balanced feature contributions appropriate for ecological index derivation, with low-risk areas reliably identified.

Patient-level risk scores were aggregated to the census block group and the resulting BVI distribution across Dallas County (**Figure S1**, map panel) shows high-risk concentrations in central Dallas, DeSoto, and Lancaster versus low-to-moderate risk in suburban Carrollton and Richardson, aligning with known geospatial signatures of socioeconomic disadvantage.

### External Validation of BVI (cross sectional):

Table 2 shows the key characteristics of our validation sample. After adjusting for age and sex, higher BVI was associated with lower MoCA scores in the combined sample (β=−0.35, 95% CI (−0.63, −0.07), R^2^=0.06) and in DHS (β=−1.06, 95% CI (−1.41, −0.71), R^2^=0.19) but not in the baseline ADC sample (β=0.14, 95% CI (−0.26, 0.53)). Using the top quintile cut-off (≥ 0.5282), those in the highest risk group had lower MoCA scores (Cohen’s d=−0.33, p<0.001) in the combined analysis and only in DHS. Participants residing in block groups with higher BVI were also more likely to have cognitive impairment in the DHS cohort only (OR: 1.55 95% CI (1.04–2.33), p=0.0335). These results are shown in Table 3 and **Figure S2 and S3** in supplemental materials.

### Construct Validity and Incremental Validity:

BVI demonstrated strong positive correlations with both SVI (r = 0.723, R^2^ = 0.522, p < 0.001) and ADI (r = 0.700, R^2^ = 0.491, p < 0.001), indicating substantial shared variance with established measures of neighborhood disadvantage. These results are shown in **Figure S4** in supplemental materials. SVI showed an association with MoCA scores (β = −0.42, 95% CI(−0.59, −0.25)) but not ADI (β = −0.14, 95% CI (−0.34, 0.05). Neither ADI nor SVI were associated with cognitive impairment risk in the combined or individual cohort analyses using the top quintile cutoffs. (Table 3) The base model including ADI, SVI, age, gender, and cohort accounted for 7.7% of variance in MoCA scores (R^2^ = 0.077). Addition of BVI to this model produced a statistically significant increment in explained variance (ΔR^2^ = 0.004, p = 0.019) suggesting BVI has unique incremental contribution to cognition beyond traditional measures.

### Longitudinal analysis in ADC cohort:

**Figure 2:**
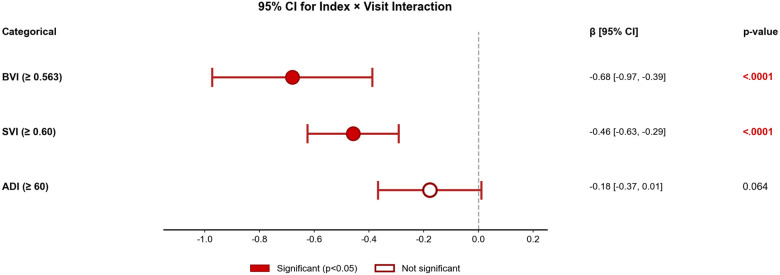
Forest Plot of Index × Visit Interactions Predicting Longitudinal Cognitive Decline. Mixed-effects models examined whether neighborhood vulnerability indices predicted differential rates of MoCA decline over time. The interaction coefficient (β) represents the additional change in MoCA score per visit for high-risk compared to low-risk individuals. Negative values indicate accelerated cognitive decline in the high-risk group. BVI demonstrated the largest effect (β = −0.68), followed by SVI (β = −0.42), while ADI did not reach statistical significance (β = −0.14, p = 0.146). Filled circles indicate significant interactions (p < 0.05); open circles indicate non-significant interactions. Error bars represent 95% confidence intervals. Models adjusted for age and sex with random intercepts for participants. Abbreviations: BVI, Brain Vulnerability Index; SVI, Social Vulnerability Index; ADI, Area Deprivation Index; CI, confidence interval.

CART analysis identified an optimal BVI threshold of ≥0.563 that maximally discriminated longitudinal cognitive trajectories. This threshold classified 152 participants (17.6%) as high-risk in the ADC longitudinal cohort. Linear mixed-effects models revealed that those with an address in the block group in the high-risk BVI demonstrated accelerated cognitive decline (Figure 2). High-risk individuals (BVI ≥0.563) exhibited a significantly faster rate of MoCA decline compared to low-risk individuals (β = −0.68, 95% CI [−0.97, −0.39], p < 0.0001). This translates to an additional 0.68-point decline per visit among high-risk individuals, representing 2.5-fold faster cognitive decline compared to the low-risk group. SVI but not ADI was associated with accelerated decline in MoCA scores (SVI: β = −0.42, 95% CI (−0.59, −0.25), p < 0.0001; ADI: β = −0.14, 95% CI (−0.34, 0.05), p = 0.146; Figure 2).

## Discussion

Although prior evidence supports the role of SDOH in dementia risks,^59–61^ this study addresses a persistent gap in this field: the absence of a clinically anchored, brain-specific geospatial risk index capable of identifying high-risk communities for cognitive impairment using routinely available data at population scale. Leveraging a ML framework that integrates EHR-derived clinical outcomes with multidimensional neighborhood-level SDOH from the CVC, we developed and externally validated the BVI.^62,63^ Across two independent geographically overlapping cohorts with complementary epidemiological designs, BVI demonstrated consistent associations with concurrent cognitive performance and with the trajectory of cognitive decline over time. BVI was equivalent to or outperformed established SDOH neighborhood indices in detecting at-risk communities but offered the advantage of being an absolute risk discriminator, which allows for improved clinical implementation at the patient level. Prior risk indices provide relative ranking of geographic areas and not actual patient-level predictive probabilities. BVI fills this gap in applying SDOH in clinical workflows.

BVI followed an outcome-anchored derivation that ensured that the features retained in the model (and their corresponding assigned weights) were empirically linked to the health endpoint of interest rather than applied post hoc to a domain for which they were not designed. The strong convergent correlations between BVI and both ADI and SVI confirm that BVI captures the same broad landscape of neighborhood disadvantage, while its incremental predictive performance in longitudinal analyses confirms meaningful discriminant validity beyond these existing measures.

Neighborhood-level chronic disease burden and educational attainment emerged as the dominant predictive features in the BVI model, consistent with the growing literature identifying cardiometabolic risk factors and cognitive reserve as two of the most modifiable upstream determinants of ADRD.^4,62^ The inclusion of environmental exposures (air quality, greenspace), behavioral risk factors (alcohol use disorder prevalence), and social infrastructure indicators (connectivity, walkability, literacy) reflects the multidimensional nature of neighborhood influence on brain health and represents a meaningful expansion beyond the financial and structural variables that dominate existing indices.^64^ This breadth of domain coverage is a core feature of the CVC framework upon which BVI is built, and it may explain BVI’s ability to capture cognitive-specific neighborhood risk that generic indices may miss.

The longitudinal findings carry translational significance. The ability of a passively derived, location-based index to discriminate meaningfully different rates of cognitive decline without requiring clinical measurement underscores the scalability of BVI for proactive population brain health surveillance.^60^ A data-driven risk stratification threshold, identified through CART analysis, provides a clinically interpretable decision boundary that can be operationalized in health system workflows using routinely available residential address data, a key prerequisite for real-world deployment at scale.

This study has multiple strengths. The two-stage hybrid design separating BVI derivation in a large retrospective EHR case-control study from external validation in two independent cohorts rigorously protects against overfitting and ensures that performance metrics reflect true out-of-sample generalizability rather than in-sample model fit, a standard that prior risk scores may have not met. The dual-cohort approach, rarely employed in prior geospatial ADRD research, provides a more complete and honest characterization of BVI’s performance envelope than single-cohort validation alone. BVI incorporates a neurologically informed, multidimensional set of SDOH domains spanning environmental exposures, cognitive enrichment, health behaviors, and material deprivation. This breadth aligns with the emerging scientific consensus that ADRD risk is shaped by a constellation of upstream social, environmental, and behavioral factors rather than any single deprivation dimension.^65^ Balancing this high dimensionality, we implemented the combination of LASSO regularization and SHAP interpretability analyses providing methodological rigor and clinical interpretability.^51,66^ The former controls for multicollinearity and overfitting in a high-dimensional feature space; the latter enables feature-level explanation of neighborhood risk contributions, supporting the kind of mechanistic understanding needed to translate index findings into targeted community interventions.

This study also has multiple limitations. The key limitation is its geographic narrowness. CVC-derived SDOH indicators were developed and calibrated within the North Texas region, and the performance of the BVI in other geographic contexts where the prevalence, intercorrelation, and health impact of neighborhood risk factors may differ substantially remains untested. However, given that most of the factors included in BVI are derived from national data, it is feasible and highly possible to generate a national-level BVI that is generalizable to all states.

Case ascertainment in the derivation cohort relied on ICD-based EHR documentation and ADRD-specific prescriptions, both of which are subject to systematic underdiagnosis.^67,68^ Differential diagnostic intensity across racial and ethnic groups is well documented in the ADRD literature and may have introduced ascertainment bias into the development sample, potentially influencing which neighborhood features carried the greatest predictive weight.^69^ However, our ability to validate BVI in a rigorous research context unreliant on EHR measurement bias suggests that BVI is reliable despite this constraint. As a geolocation-level index, the BVI is inherently subject to the ecological fallacy: block-group-level risk scores reflect average neighborhood exposure and cannot capture within-neighborhood heterogeneity or individual-level social circumstances that may deviate substantially from the group median.^70^ Accordingly, BVI should complement and not replace individual-level social risk screening in clinical encounters, consistent with best practices. Nevertheless, BVI is more precise than generic SDOH indices given its included patient-level risk derived from medical encounters. Finally, generalization to individuals outside of the demographic and clinical distribution of our development or validation cohorts should be done with caution given the inherent variability of cognitive impairment risk by age, sex, and race.

## Conclusion

This study demonstrates that a clinically anchored, machine learning-derived geospatial index can identify communities and individuals at elevated risk for cognitive impairment and accelerated cognitive decline using routinely available health system and publicly accessible SDOH data. BVI provides a scalable, brain-specific, equity-focused neighborhood risk tool that outperforms existing generic deprivation indices in predicting longitudinal cognitive outcomes across both community-based and clinical settings. By enabling passive, address-based risk stratification at population scale, BVI offers a practical instrument for guiding targeted prevention, early identification, and equitable allocation of resources toward communities bearing the greatest burden of neighborhood-attributable risk for cognitive impairment.

## Supplementary Files

This is a list of supplementary files associated with this preprint. Click to download.

- supplementmaterials.pdf

## Figures and Tables

**Table 1.** Characteristics of Study Population Used for the Development of the Brain Vulnerability Index Source: Authors’ own analyses.

	Overall	EHR-Evidence for Cognitive Impairment= No	EHR-Evidence for Cognitive In
	N=231,630	N=192,060	N=39,570
**Age**	61.87 (14.91)	66.70 (18.61)	60.87 (13.82)
**Sex**			
*Female*	126,894 (54.78%)	105,605 (54.99%)	21,289 (53.80%)
*Male*	104,736 (45.22%)	86,455 (45.01%)	18,281 (46.20%)
**First Race**			
*American Indian*	643 (0.28%)	528 (0.27%)	115 (0.29%)
*Asian*	7,740 (3.34%)	6,347 (3.30%)	1,393 (3.52%)
*Black or African American*	72,028 (31.10%)	57,640 (30.01%)	14,388 (36.36%)
*Other Pacific Islander*	727 (0.31%)	605 (0.32%)	122 (0.31%)
*Unkown*	21,158 (9.13%)	20,291 (10.56%)	867 (2.19%)
*White*	129,334 (55.84%)	106,649 (55.53%)	22,685 (57.33%)
**Ethnicity**			
*Hispanic*	90,241 (38.96%)	77,060 (40.12%)	13,181 (33.31%)
*Non-Hispanic*	118,723 (51.26%)	93,232 (48.54%)	25,491 (64,42%)
*Unknown*	22,666 (9.79%)	21,768 (11.33%)	898 (2.27%)
**Smoking Status**			
*Ever*	67,571 (29.17%)	50,613 (26.35%)	16,958 (42.86%)
*Never*	164,059 (70.83%)	141,447 (73.65%)	22,612 (57.14%)
**Marital Status**			
*Divorced or Legally Separated*	27,371 (11.82%)	21,389 (11.14%)	5,982 (15.12%)
*Married, Common Law or Significant Other*	78,834 (34.03%)	68,250 (35.54%)	10,584 (26.75%)
*Single*	81,640 (35.17%)	65,084 (33.89%)	16,376 (31.38%)
*Unknown*	28,161 (12.16%)	27,415 (14.27%)	746 (1.89%)
*Widowed*	15,804 (6.82%)	9,922 (5.17%)	5,882 (14.86%)
Charlson Comorbidity Index (Age-Adjusted)	0.61 (1.58)	2.00 (2.56)	0.32 (1.09)
			
**SDOH (Average Index/Indicator Value)** **			
*Community Vulnerability Index)*	0.55 (0.27)	0.54 (0.27)	0.59 (0.26)
*Household Essentials Index*	0.54 (0.27)	0.53 (0.27)	0.58 (0.26)
*Empowered People Index*	0.51 (0.26)	0.5 (0.26)	0.55 (0.24)
*Equitable Communities Index*	0.61 (0.24)	0.61 (0.24)	0.64 (0.23)
*Good Health Index*	0.48 (0.25)	0.47 (0.25)	0.52 (0.25)
**Healthcare Utilization, 1 year prior to diagnosis**			
*Primary Care Visits*	*136085 (0.59±3.29 per patient)*	*86758 (0.45±2.64 per patient)*	*49327 (1.25±5.37 per patient)*
*ED Visits*	*1505 (0.01±0.08 per patient)*	*868 (0±0.07 per patient)*	*637 (0.02±0.13 per patient)*
*Hospital Admissions*	*276 (0±0.03 per patient)*	*50 (0±0.02 per patient)*	*226 (0.01±0.08 per patient)*
*Mental Health Encounters*	*1250 (0.01±0.24 per patient)*	*370 (0±0.13 per patient)*	*880 (0.02±0.5 per patient)*
*Neurology Encounters*	*5732 (0.02±0.51 per patient)*	*1951 (0.01±0.29 per patient)*	*3781 (0.1±1.04 per patient)*
*Rheumatology Encounter*	*1464 (0.01±0.21 per patient)*	*911 (0±0.18 per patient)*	*553 (0.01±0.3 per patient)*
*Gastroenterology Encounter*	*3928 (0.02±0.36 per patient)*	*2136 (0.01±0.27 per patient)*	*1792 (0.05±0.64 per patient)*
*Pulmonology Encounter*	*1921 (0.01±0.23 per patient)*	*959 (0±0.18 per patient)*	*962 (0.02±0.42 per patient)*

* Student t-test performed for mean difference between Alzheimer’s Dementia Status

** Higher the value, higher the vulnerability

**Table 2.** Key Characteristics of External Validation Study Participants Source: Authors’ own analyses.

Characteristic	ADC (N=863)	DHS (N=532)	Combined (N=1,395)
**Demographics**			
Age, years, mean (SD)	70.1 (8.3)	60.3 (10.6)	66.4 (10.4)
Female, n (%)	440 (51.0)	322 (60.5)	762 (54.6)
**Race/Ethnicity, n (%)**			
White	713 (82.6)	161 (30.3)	874 (62.7)
African American	139 (16.1)	310 (58.3)	449 (32.2)
Other	11 (1.3)	61 (11.5)	72 (5.2)
**Cognitive Measures**			
MoCA score, mean (SD)	22.3 (5.7)	22.1 (4.4)	22.2 (5.2)
Cognitive impairment, n (%)	551 (63.9)	156 (29.3)	707 (50.7)
**Impairment Category, n (%)**			
Normal cognition	312 (36.2)	376 (70.7)	688 (49.3)
Mild cognitive impairment	218 (25.3)	153 (28.8)	371 (26.6)
Dementia	333 (38.6)	3 (0.6)	336 (24.1)
**Neighborhood Indices**			
BVI (block group), mean (SD)	0.45 (0.07)	0.48 (0.08)	0.46 (0.08)
SVI, mean (SD)K	0.37 (0.28)	0.54 (0.30)	0.44 (0.30)
ADI national percentile, mean (SD)K	38.7 (25.8)	56.5 (26.1)	45.7 (27.3)

Abbreviations: ADC = Alzheimer’s Disease Center; DHS = Dallas Heart Study; SD = standard deviation; MoCA = Montreal Cognitive Assessment; BVI = Brain Vulnerability Index; SVI = Social Vulnerability Index; ADI = Area Deprivation Index.

SVI available for N=772 (ADC) and N=496 (DHS); ADI available for N=771 (ADC) and N=495 (DHS).

**Table 3.** Cross-sectional Association of Neighborhood Vulnerability Indices with Cognitive Outcomes Source: Authors’ own analyses.

Characteristic	ADC (N=863)	DHS (N=532)	Combined (N=1,395)
**MoCA scores**			
Unadjusted, β (95% CI)	0.14 (−0.25, 0.54)	−1.36 (−1.72, −1.01)***	−0.47 (−0.76, −0.19)**
Adjusted, β (95% CI)	0.14 (−0.26, 0.53)	−1.06 (−1.41, −0.71)***	−0.35 (−0.63, −0.07)*
R^2^	0.028	0.191	0.061
**Risk Stratification (BVI ≥0.5282)**			
High risk, n (%)	119 (13.8)	159 (29.9)	278 (19.9)
MoCA difference, unadjusted	−0.63 (−1.73, 0.47)	−2.73 (−3.52, −1.93)*	−1.72 (−2.42, −1.02)*
MoCA difference, adjusted	−0.82 (−1.91, 0.28)	−2.21 (−2.97, −1.45)***	−1.55 (−2.23, −0.87)***
Cohen’s d	−0.11	−0.64	−0.33
**Cognitive Impairment risk for top quintile**			
**BVI (≥0.5282)**			
High risk, n (%)	122 (14.1)	159 (29.9)	281 (20.1)
Unadjusted OR (95% CI)	0.92 (0.62–1.37)	1.61 (1.08–2.40)*	1.22 (0.92–1.63)
Adjusted OR (95% CI)	1.07 (0.71–1.60)	1.55 (1.04–2.33)*	1.27 (0.95–1.70)
**SVI (≥0.60)**			
High risk, n (%)	186 (24.1)	231 (46.6)	417 (32.9)
Unadjusted OR (95% CI)	1.03 (0.73–1.44)	1.39 (0.94–2.06)	1.17 (0.91–1.52)
Adjusted OR (95% CI)	1.10 (0.77–1.56)	1.39 (0.93–2.06)	1.20 (0.93–1.57)
**ADI (≥60)**			
High risk, n (%)	174 (22.6)	236 (47.7)	410 (32.4)
Unadjusted OR (95% CI)	0.98 (0.69–1.39)	1.45 (0.98–2.14)	1.17 (0.90–1.52)
Adjusted OR (95% CI)	1.12 (0.78–1.60)	1.48 (0.99–2.20)	1.25 (0.96–1.63)

Abbreviations: BVI = Brain Vulnerability Index; SVI = Social Vulnerability Index; ADI = Area Deprivation Index; OR = odds ratio; CI = confidence interval.

Values are regression coefficients from the general linear models (GLM) with MoCA score modeled as a continuous dependent variable; BVI entered as a continuous standardized BVI along with study indicator in combined analysis.

Adjusted for age and sex (study indicator added in combined analysis).

* p<0.05,

** p<0.01,

*** p<0.001

## Data Availability

Data is Available upon request

## Abbreviations

ADC: Alzheimer’s Disease Center
DHS: Dallas Heart Study
SD: standard deviation
MoCA: Montreal Cognitive Assessment
BVI: Brain Vulnerability Index
SVI: Social Vulnerability Index
ADI: Area Deprivation Index
